# Nucleoside Hydrolase NH 36: A Vital Enzyme for the *Leishmania* Genus in the Development of T-Cell Epitope Cross-Protective Vaccines

**DOI:** 10.3389/fimmu.2019.00813

**Published:** 2019-04-16

**Authors:** Clarisa Beatriz Palatnik-de-Sousa

**Affiliations:** ^1^Institute of Microbiology Paulo de Góes, Federal University of Rio de Janeiro, Rio de Janeiro, Brazil; ^2^Faculty of Medicine, Institute for Research in Immunology, University of São Paulo, São Paulo, Brazil

**Keywords:** visceral leishmaniasis vaccines, cutaneous leishmaniasis vaccines, nucleoside hydrolases, multi-epitope vaccines, Leishmune® vaccine

## Abstract

NH36 is a vital enzyme of the DNA metabolism and a specific target for anti-*Leishmania* chemotherapy. We developed second-generation vaccines composed of the FML complex or its main native antigen, the NH36 nucleoside hydrolase of *Leishmania (L.) donovani* and saponin, and a DNA vaccine containing the NH36 gene. All these vaccines were effective in prophylaxis and treatment of mice and dog visceral leishmaniasis (VL). The FML-saponin vaccine became the first licensed veterinary vaccine against leishmaniasis (Leishmune®) which reduced the incidence of human and canine VL in endemic areas. The NH36, DNA or recombinant protein vaccines induced a Th1 CD4^+^IFN-γ^+^ mediated protection in mice. Efficacy against VL was mediated by a CD4^+^TNF-α T lymphocyte response against the NH36-F3 domain, while against tegumentary leishmaniasis (TL) a CD8^+^ T lymphocyte response to F1 was also required. These domains were 36–41 % more protective than NH36, and a recombinant F1F3 chimera was 21% stronger than the domains, promoting a 99.8% reduction of the parasite load. We also identified the most immunogenic NH36 domains and epitopes for PBMC of active human VL, cured or asymptomatic and DTH^+^ patients. Currently, the NH36 subunit recombinant vaccine is turning into a multi-epitope T cell synthetic vaccine against VL and TL.

## Introduction

In recent years, nucleoside hydrolases (NHs) of protozoa emerged as strong phylogenetic markers (1, 2) and as vital protagonists of pathways for parasite replication (3–5). Moreover, since they are also involved in the establishment of early infection (3–5), they can be considered new targets for drug development (5–8) and excellent candidates for the pathogen recognition by adaptive immune responses (9–15).

Protozoa, fungi and bacteria obtain their purines after the cleavage of exogenous DNA through the enzymatic activity of NHs (3, 16). These enzymes, which are crucial for pathogen replication, are expressed in the early stages of infection. In contrast, nucleoside hydrolases are absent from mammal cells, which are capable of synthesize the purines and pyrimidines needed for their own DNA replication (3). Consequently, NHs are excellent targets for selective toxicity against pathogens.

Additionally, these enzymes, which are expressed at the initial stages of infection of many pathogens, are very immunogenic and share high levels of identity in their amino acid sequences, therefore, generation of protection against them would prevent the early replication of many different microorganism (13, 15, 16). Hence, vaccines developed against NHs of one pathogen could determine strong protection against infections caused, not only by the homologous strain, but also by different strains or by pathogens of other related or non–related taxonomic groups (9–14). In this sense, NHs become excellent candidates for the development of universal vaccines, which are considered one of the highest priorities for the effective control of epidemics.

In this review, we will discuss the potential use of NHs as targets of the chemotherapy against leishmaniasis. Furthermore, we will consider the use of immucillins, which are synthetic analogs of nucleosides that inhibit the enzymatic function of NHs and cure leishmaniasis through a bivalent effect: the direct toxicity against the *Leishmania* parasites and the stimulation of a Th1 immune response of the host. Additionally, we will show that the main antigen of the first licensed anti-*Leishmania* vaccine called Leishmune® (17–22), is a NH (23) that evolved to a DNA (24) and recombinant protein vaccine. NH is now the focus of the development of a synthetic multiepitope T-cell vaccine against leishmaniasis (10–14).

## Nucleoside Hydrolases Are Key Enzymes for the Replication of Parasitic protozoa, Yeast and Bacteria

To establish an infection in human and mammal reservoirs, pathogens must be able to synthesize their own DNA efficiently and replicate intensively. Mammal cells use the *de novo* pathway to obtain purines (16) in their purinosomes (25). In contrast, fungi, bacteria (16), and parasitic protozoa like *Plasmodium* (26, 27), *Entamoeba hystolytica* (28, 29), *Giardia lamblia* (30), *Toxoplasma gondii* (31, 32), *Trichomonas vaginalis* (33), *Trypanosoma cruzi* (34), *Trypanosoma brucei* (35–37), and *Leishmania* (38, 39), are purine auxotrophs, which lack the *de novo* metabolic route and obtain their purines and pyrimidines from imported DNA through salvage pathways.

All these pathogens import DNA from exogenous sources and nucleotidases/nucleases release nucleotides at the level of the plasma membrane. These nucleotides are de-phosphorylated and transported into cells as nucleosides (40). Alternatively, nucleosides are carried into cells by specific transporters (41). Once inside the cells, nucleoside hydrolases (39) or nucleoside phosphorylases (PNP) (42) in the cytoplasm, release the purines from the nucleosides, which can further be used by the pathogen for the synthesis of its DNA.

Since the enzymatic function of NHs is so mandatory for pathogens and because they are absent from mammals cells, they become excellent targets for selective chemotherapy.

## Nucleoside Hydrolases Are Phylogenetic Markers of the *Leishmania* Genus

As expected for their essential role in the replication of intracellular parasites, NHs are present in all the studied species of the *Leishmania* genus (13, 15, 39, 43, 44). In fact, NHs that share high levels of homology in their amino acid sequences to NH36 have been described in *L. (L.) major* (95–96%), *L. (L.) mexicana* (93%), *L. (L.) infantum chagasi* (99%), *L. (L.) infantum* (99%), *L. (L.) tropica* (97%), *L. (V.) braziliensis* (84%), *L. (L.) amazonensis* (93%), *L. (V.) guyanensis* (84 %), and *L. (L.) aethiopica* (30 %) (12, 13, 15, 43, 44). This high level of identity justifies that fact that these enzymes are considered very relevant as taxonomic and phylogenetic markers of the genus *Leishmania*. Accordingly, two nucleoside hydrolases were chosen, together with three other metabolic enzymes, as the basis of a profound analysis of the taxonomy and phylogeny of the *L. (L.) donovani* complex. This analysis was performed using multilocus sequence typing (2). These enzymes are dimeric and encoded by a single gene. Actually, the presence of NH1 and NH2 was confirmed in 17 isolates of *L. (L.) infantum* and in 20 strains of *L. (L.) donovani* from the World Health Organization Bank. Confirming their status as conservative markers of the *Leishmania* genus, the NH1 and NH2 genes were expressed by the 1–2 allele forms in the strains of *L. (L.) infantum* and by the 3–6 alleles in the strains of *L. (L.) donovani* (2).

## Nucleoside Hydrolases Are Targets of Chemotherapeutic Nucleoside Analogs

NHs are attractive targets for the chemotherapy of leishmaniasis. The inhibition of the enzymatic functions of NHs would impair the parasite growth with no damage to the mammal host cells. In a recent work we investigated the potential inhibitory action of synthetic nucleoside analogs called immucillins on *Leishmania* replication *in vitro* and on the enzymatic function of the nucleoside hydrolase of *Leishmania (L.) donovani* NH36 (5).

Immucillins are synthetic deazapurine iminoribitols which have been described as inhibitors of the *in vitro* activity of the nucleoside hydrolases of *Crithidia fasciculata* (39), *Leishmania (L.) major* (39), and *Trypanosoma brucei brucei* (36); however, with no record of anti-parasite activity. Lately, second and third generation immucillins showed inhibitory activity against PNP (45) and were chemotherapeutic against malaria of monkeys infected with *Plasmodium falciparum* (26).

We recently demonstrated that NH36 of *L (L.) donovani* is a non-specific nucleoside hydrolase, which has adenosine and inosine as favored substrates and develops good catalytic activity against uridine and cytidine pyrimidine nucleosides (5). *L. (L.) donovani* parasites cease to grow after purine starvation, but for adaptation purposes cells suffer a proteome re-modeling and they upregulate purine transport, salvage of purines metabolism and their correspondent proteins (46). The proteomes of purine-starved and purine repleted parasites were recently compared. After 48 h of purine starvation, the expression of the purine-specific nucleoside hydrolase (putative inosine-adenosine-guanosine-nucleoside hydrolase IAGNH, LinJ.292910) and the 6-hydroxipurine nucleoside hydrolase (inosine-guanosine-nucleoside hydrolase, IGNH, LinJ.14.0130) were enhanced. In contrast, the non-specific NH (LinJ178.1570) showed no significant change or even a decreased expression (46).

Inhibition of the replication of promastigotes of *L. (L.) infantum chagasi* and *L. (L.) amazonensis in vitro* was achieved after co-incubation with the first generation Immucilin A (IA) and Immuclin H (IH), second-generation DadME-Immucilin H (DIH) and fourth generation SerMe-Immucilin H (SMIH), SerMe-Immucilin G (SMIG), and SerMe-Immucilin A (SMIA), at nanomolar to micromolar concentrations (5). Their efficacies on both species of *Leishmania* were similar, but DadME-Immucilin G (DIG) was more potent to inhibit *L. (L.) infantum chagasi*, and SMIA against *L. (L.) amazonensis*. IA, IH and SMIH were the most potent growth inhibitors of both parasites (5). Additionally, we proved that IA, which is a synthetic analog of adenosine, inhibited the *Leishmania* replication in a chemically defined medium deprived of nucleosides sources, and that the addition of adenosine reverted this effect (5).

Furthermore, we demonstrated that the inhibition of *Leishmania* replication by treatment with IA and IH was mediated by the inhibition of the NH36 enzymatic activity (5). In addition, both immucillins and SMIH also inhibited the replication of intracellular amastigotes of *L. (L.) infantum chagasi* causing no damage to the macrophage host cells. Additionally, the treatment with IA and IH promoted the swelling and morphological alteration of promastigotes while SMIH caused intense vacuolization and kinetoplast damage (5).

Our results confirmed that NH36 is a vital enzyme for *Leishmania* replication and therefore, it is reasonable to assume that it is also, an excellent target for save chemotherapy against leishmaniasis.

## Drugs That Target Nucleoside Hydrolases Are Also Immunogenic

The *in vivo* chemotherapeutic potential of the IA, IH and SMIH immucillins was further assayed in BALB/c mice infected with *L. (L.) infantum chagasi* amastigotes and compared with that of the golden standard drug Glucantime® (6). Visceral leishmaniasis is a chronic and frequently lethal protozoan disease against which no human vaccine is available and current treatment is reduced to a few highly toxic drugs administered intravenously, thus requiring hospitalization.

Among the immucillins, IA induced the strongest reduction of parasite load (89%), followed by IH (85%) and SMIH (85%). Treatment with immucillins also determined high titers of anti-NH36 IgG2a antibodies and a gain in corporal weight, further the immucillins prevented the weight increase in the liver and spleen detected in infected controls, which exhibited the highest IgG1 anti-NH36 response (6). All immucillins, most notably IA, promoted in splenocytes a higher secretion of IFN-γ and TNF-α to the supernatants than the infected controls. In contrast, the IL-10 secretion was observed mainly in the infected untreated controls.

This remarkable significant increase of secretion from the pro-inflammatory cytokines after immucillin treatment could be due to the killing of parasites and the recovery of the Th1 immune response of the host, or to a direct immunogenic effect of the drugs. Therefore, to clarify this question we treated normal uninfected mice and observed that the secretion of IFN-γ and TNF-α from splenocytes, in response to NH36, was enhanced more after therapy with immucillins than with Glucantime®, which in contrast increased the IL-10 secretion. In addition, IA increased the frequencies of CD4^+^ T and CD19^+^ B lymphocytes, while SMIH increased only the CD19^+^ B cells counts (6). The IA and IH immucillins were also therapeutic in *L. (L.) infantum chagasi* infected mice and hamsters showing no toxicity. In contrast, the treatment with Glucantime® promoted high levels of urea, creatinine, aspartate aminotransferase (GOT) and alanine aminotransferase (GPT) in mice. These experimental data indicated that IA induced the strongest therapeutic effect on visceral leishmaniasis probably through the combined toxicity against the parasite and the induction of a Th1 immune response of the host (6).

## The NH36 of *Leishmania (L.) donovani* Is the Main Antigen of the Second-Generation Leishmune® Vaccine

Visceral leishmaniasis is a highly endemic and epidemic protozoan vector-borne disease which causes 400,000 new annual cases and it is frequently lethal if untreated soon after the onset of symptoms. Only a few drugs are used for the chemotherapy, which is still highly toxic. Therefore, the development of a preventive specific vaccine for the epidemiological control of VL is considered urgent (47). VL is a human anthroponosis caused by *L. (L.) donovani* in India, Asia and East Africa, and a canid zoonosis caused by *L. (L.) infantum chagasi* in the Americas and by *L. (L.) infantum* in the Middle East, Central Asia, China and the Mediterranean (48). Ninety percent of the world incidence of the disease is concentrated in Bangladesh, India, Nepal, Sudan, Ethiopia and Brazil. Although four vaccines against canine VL have already been licensed (Leishmune® (17–22), Leishtec® (49), CaniLeish® (50), and Letifend® (51), no vaccine against the human disease is yet available.

In the search for the most immunogenic fraction of the promastigotes of *L. (L.) donovani* (Sudan), we described, in the 80's, the Fucose Mannose Ligand (FML), which is composed of several protein bands and inhibited *in vitro* the penetration of promastigotes (52) and amastigotes into the macrophage host cells (53) in a species-specific manner (54). The FML is strongly immunogenic and antigenic in mice and rabbits and most of the monoclonal antibodies raised against it react with its 36 kDa glycoproteic component (53). The FML was used with success in the specific serodiagnosis of human (55) and dogs (56) with visceral leishmaniasis, and for VL control of blood transfusion (57) and, together with its 36 kDa component (58), induced an effective protective vaccination in mice and hamsters against *L. (L.) donovani* infection (59, 60). Saponins, mainly those of *Quillaja saponaria* Molina, showed the best performance as adjuvants of these vaccine formulations (61, 62). The FML-saponin vaccine induced strong prophylactic protection in a Phase II trial performed on dogs challenged with *L. (L.) donovani* (21), and in two Phase III trials in endemic areas of Brazil, where it showed 76–80 % of vaccine efficacy (21, 63, 64). These field trials were performed in an area of high infective pressure, where cases of VL were considered animals that died of the disease, or that exhibited symptoms of VL and had parasitological confirmation. The vaccine promoted strong increases of anti-FML antibodies (63, 64), mainly of the IgG2 subtype (65) and intradermal reaction of the *L. (L.) donovani* promastigote antigen, while significantly reduced the number of deaths, as well as the clinical and parasitological sings of the disease (63, 64). In fact, protection lasted for at least 3.5 years (64).

The FML-saponin vaccine, formulated with an increased dosage of adjuvant, was also effective in the therapy of canine visceral leishmaniasis in endemic areas (66). After immunotherapy, no deaths were registered among infected and treated dogs. These animals showed increased anti-FML IgG2 titers and higher percent of positive intradermal response to the leishmanial lysate (IDR) and reduced symptomatology. Furthermore, their normal proportions of CD4^+^ T lymphocytes and CD21^+^ B lymphocytes, suggested their non-infectious condition. Also, their percentages of CD8^+^ T cells increased, as was expected after treatment with *Quillaja saponaria* saponin (66).

After that investigation, the FML-saponin vaccine started to be manufactured by Fort-Dodge-Pfizer-Zoetis, and was licensed in Brazil in 2003, as the first worldwide anti-*Leishmania* vaccine, called Leishmune® (21). The immunogenicity of Leishmune® was proven in 550 dogs of seven Brazilian towns in endemic areas (17). The vaccine showed to be safe (18), sustained the immunogenicity described for the FML-saponin formulation, and promoted a high anti-FML antibody and IDR response, with increased frequencies of CD8^+^ and CD21^+^ T cells, and sustained levels of CD4^+^ T cells. Leishmune® also determined a reduction in the number of deaths and symptoms (17). The vaccine was further useful in the therapy of experimental (67) and naturally acquired (68) visceral leishmaniasis. It reduced deaths and determined the clinical and parasitological cure of VL. In addition, PCR analysis revealed that a combined treatment with the vaccine and Amphotericin was necessary to achieve the sterile cure (68). Since then, many investigations disclosed the higher efficacy of Leishmune® to Leishtec® and/ or LBsap vaccines (69, 70), its stronger immunogencity and pro-inflammatory response (71–73) and its capability of raising the frequencies of CD8 T cells secreting-IFN-γ (74).

This is an inherent property of the *Quillaja saponaria* adjuvant QS21, which composes the vaccine (75). QS21 saponin is a composed by a triterpene nucleus to which one glycidic moiety is attached to C-3 and a combined glycidic and normonoterpene moiety is attached to C-28. The normonoterpene moiety has been associated to the promotion of a cytotoxic CD8- T cell response (75). Additionally, QS21 has an aldehyde attached to C4, which is involved in the triggering of the Th1 response (75).

Healthy dogs vaccinated with Leishmune® and exposed in endemic areas showed increased antibody and IDR responses but reduced mortality compared to unvaccinated controls (17). Remarkably, dogs vaccinated with Leishmune® not only offer fewer parasites (19) but also, their anti-FML antibodies block the further development of the parasite inside the guts of the to the insect vector (20, 21). These two effects combined defined Leishmune® as a transmission blocking vaccine (20, 21) and this explains why it reduces the incidence of both, the canine and human diseases as predicted (76) interrupting the epidemics (22). The decreased offer of parasites to sand fly vectors showed in vaccinated dogs, is associated to the absence of *Leishmania* antigen in skin, and DNA, in blood and lymph nodes (19).

In addition, regarding the Leishmune® vaccine, the western blot analysis of the FML antigen disclosed that the 36 KDa component was specifically recognized by the sera of human patients of VL (77) and it was therefore, the main antigen of the Leishmune®. The molecular cloning of the 36 kDa protein identified that it is a nucleoside hydrolase of *L. (L.) donovani*, which we denominated NH36 (23).

Leishmune® was the first licensed vaccine against canine leishmaniasis. Its license was obtained in Brazil in 2003 (19–22). The current other three commercial vaccines: Leishtec® (49), Canileish® (50), and Latifend (51) were licensed after Leishmune® but also use as adjuvants, saponins of *Quillaja saponaria*.

Leish-Tec®, contains the recombinant protein A2 of amastigotes of *L. (L.) donovani* and showed to be protective against VL in assays with experimental challenges in the mice (78–80), Rhesus monkeys (81), and beagle dog models (82). The license for commercialization of the Leishtec® vaccine was approved in 2008. The first field trial assay of Leishtec®, was published in 2016 and was performed in an area of low infective pressure (49). In this area, cases of VL where defined, not by deaths or clinical symptoms with parasite confirmations, as it was used for the Leishmune® vaccine (21, 63, 64), but, instead, by seropositivity confirmed by at least one of three parasitological assays, or by adding xenodiagnoses to parasite assays (49). Using these less severe criteria, the authors reported 71.4% vaccine efficacy, based on the parasitological definition of cases and 58.1%, by adding xenodiagnoses (49). In agreement with that, when a field trial of Leishtec® was performed in a high infective pressure area, a lower reduction in the rate of canine infection was observed (83). In fact, the incidence of VL was 42% in controls and 27% in vaccinated dogs, representing only a 35.7% of vaccine efficacy (83). In addition, protective responses were either, not generated or not maintained, in 43% of the immunized dogs that became infected and developed disease overtime. Furthermore, the levels of anti-A2 specific IgG antibodies were significantly higher in vaccine recipients than in infected control dogs confirming the immunogenicity of A2 (83).

Furthermore, a recombinant protein, named Q formed by the genetic fusion of five intracellular antigenic fragments, from the *L. (L.) infantum* acidic ribosomal proteins Lip2a, Lip2b, P0, and histone H2A (51, 84) was licensed as the Letifend® vaccine. In a recent field trial in France and Spain, the definition of a case included clinical signs confirmed by positive Elisa and the finding of parasites in bone marrow or lymph nodes through PCR or microscopic observation of smears. Based on these results the vaccine efficacy was 72% (84).

Finally, the CaniLeish® vaccine is composed of the *Leishmania (L.) infantum* Excreted Secreted Proteins (ESP) and the Purified extract of Quillaja saponaria (QA-21) also showed Th1 dominated immune response which persisted for a full year (85). A field trial performed in high endemic areas of the Mediterranean basin used PCR and parasite cultures for confirmation of infection (50). Considering the active infection, a 63% of vaccine efficacy was observed on month 24 after vaccination. Considering the VL symptoms, the vaccine efficacy was 68.4% (86). In agreement, it has been acknowledged that only two vaccines, consisting of parasite purified fractions with saponin derivative adjuvants, showed to confer significant protection against disease and death under natural conditions, the FML-QuilA (Leishmune®) in Brazil, and LiESP/QA-21 (CaniLeish®) in Europe (85).

## NHs Protein Sequences Are Very Immunogenic

Visceral leishmaniasis is characterized by fever, malaise, anemia, leukopenia, thrombocytopenia, hepatosplenomegaly, hypergammaglobulinemia, and a strong suppression of the cellular immune response (47), mainly due to an intense decrease of the CD4^+^ T helper lymphocyte population, which is responsible for the resistance to the infection (86, 87). Therefore, an effective vaccine against VL is expected to guarantee a robust Th1 CD4^+^ T cell response against the parasite infection, characterized by secretion of IL-12 by antigen-presenting cells (APCs) which promotes the increase of IFN-γ-secreting Th1 T cells (86, 88). The achievement of protection or natural resistance against VL in fact requires the induction of powerful and long-lasting Th1 parasite-specific memory responses, with multifunctional CD4^+^ T cells producing IFN-γ, IL-2, and TNF-α (10–12, 15, 89). Besides, a balance between the Th1 IFN-γ and TNF-α cytokines, the secretion of IL-17 and the regulatory cytokine IL-10 (90, 91) is needed. Furthermore, CD8^+^ T cells may contribute by secreting beneficial cytokines or with their cytotoxic activity that release amastigotes to be killed by activated monocytes (92). In fact, CD8 ^+^ T cells were recently involved in the cure or pathology of CL (93).

NH enzymes are not only present in many bacteria, fungi and parasitic protozoa species (2, 13, 16, 43, 44), in which they are expressed at early stages of infection, but they are also very immunogenic. Interestingly, NH36 is the object of many vaccine studies since it is very antigenic and immunogenic (9–15, 94, 95). These properties are based on the amino acid composition of the most immunogenic region of the NHs. Aromatic amino acids like phenyl-alanin, tryptophan and tyrosine, together with the hydrophobic amino acids leucine, isoleucine and valine, are common component of the epitopes for MHC Class I receptors on APCs (96). Phenyl-alanin and tryptophan have lateral chains, which make it easier to establish contact with the T cell receptors (97). In addition, epitopes for the MHC Class II receptors show hydrophobic residues in position 1 and 9, acidic amino acids in position 4 and basic charged amino acids in position 6 (97). Therefore, higher frequencies of hydrophobic, acidic and charged amino acids are expected to be found in the most immunogenic regions of antigens, which are enriched in T cell epitopes. In agreement to that, the NHs of *L. (L.) donovani* NH36, *L. (L.) amazonensis*, and *L. (V.) braziliensis* contain a total of 71.1, 74.1, and 71.7% of hydrophobic, charged and acidic amino acids, respectively (Table 1), which makes it reasonable to assume that they would constitute good vaccine antigens to promote the T cell immune response against the parasite.

**Table 1:** Percent of acidic, charged and hydrophobic amino acids of Nucleoside hydrolases of *Leishmania*.

**Groups**	**Amino acids**	***L. (L.) donovani* NH36 AAG02281.1**	***L. (L.) amazonensis* NH A34880**	***L. (V.) braziliensis* NH XP 1564081**
Acidic	Glu	5.1	7.2	6.4
	Asp	6.1	6.3	5.4
Charged	Lys	4.5	5.7	5.1
	Arg	5.1	3.0	4.8
Hydrophobic	Ala	9.2	12.0	8.7
	Val	12.7	10.8	10.9
	Leu	7.6	6.0	8.7
	Ile	6.1	4.5	7.7
	Pro	6.4	5.1	6.4
	Phe	3.2	3.9	2.6
	Trp	0.6	2.4	0.6
	Cys	1.6	3.6	2.2
	Met	2.9	3.6	2.2
Acidic+ charged+ hydrophobic		71.1	74.1	71.7

*The amino acid composition of NHs of L. (L.) donovani NH36, L. (L.) amazonensis and L. (V.) braziliensis were analyzed using the expasy prot param tool (https://web.expasy.org/protparam/)*.

## Development of the NH36 Vaccine Against Leishmaniasis: a Third-Generation Vaccine

Initially we studied the efficacy of a DNA vaccine containing the NH36 gene cloned in the VR1012 plasmid (24). Using a mice model we compared the efficacy of the FML and the NH36 recombinant protein (rNH36) formulated with saponin with that of the NH36 DNA vaccine, against visceral leishmaniasis due to *L. (L.) infantum chagasi*, and cutaneous leishmaniasis due to *L. (L.) mexicana* (24). The three vaccines induced the production of IgG, IgG1, and IG2a antibodies and strong IDR against *L. (L.) infantum chagasi*. The rNH36 and FML vaccine also induced enhanced IDR in mice challenged with *L. (L.) mexicana* (24).

FML and rNH36 showed efficacy of 79% in reduction of the *L. (L.) infantum chagasi* parasite load. In contrast, the VR1012-NH36 DNA vaccine was stronger and reduced the *L. (L.) infantum chagasi* parasite load by 88% and the lesion sizes by induced by *L. (L.) mexicana* (24) and *L. (L.) amazonensis* (98) by 65 and 80.4%, respectively. Remarkably, the DNA vaccine increased the frequencies of INF-γ producing CD4^+^ T cells, triggering a Th1 type immune response against NH36 (24). Our results therefore, indicated that the NH36 DNA vaccine was a very good candidate for the generation of a Th1 immune protection against several *Leishmania* species (24). In fact, this vaccine was also efficient in the immunotherapy against visceral leishmaniasis of mice (99).

In agreement with the results obtained with mice, the VR1012NH36 vaccine was used with success in prophylaxis (100) and immunotherapy of dogs infected with *L. (L.) infantum chagasi* (101). Infected dogs treated with the DNA vaccine exhibited increased sizes and proportions of IDR and frequencies of NH36-specific CD4^+^ T cell in blood. These lymphocytes recognized mainly the C-terminal peptide of NH36. On the other hand, the N-terminal of NH36 was the target of the IgG2/IgG1 enhanced antibody ratios and CD8^+^ T cell counts. Moreover, the immunotherapy with the NH36 DNA vaccine reduced the parasite load in the livers and the loss of body weight of dogs as well, increasing their survival time (101).

## Searching for the Most Immunogenic Domains of NH36

Previous concerns existed about the usage of short sequences of a protein antigen in a vaccine. In comparison to native antigens that contain PAMPs as their natural adjuvants, the recombinant proteins would be not immunogenic as they are purified and are free from PAMPs (102). Moreover, there was a general consensus that recombinant subunits would probably not retain the immunogenicity inherent to the whole protein. However, recent evidence has demonstrated that protein domains of a vaccine candidate, that concentrate its most immunogenic amino acid sequences, might impair and even exceed the protective response induced by the whole molecule (103).

Aiming to identify the most immunogenic domains of NH36, we first subcloned the NH36, which is composed of 314 amino acids, into three recombinant peptides covering all its sequence: the N-terminal, F1 (amino acids 1-103), the Central domain, F2 (amino acids 104-198), and the C-terminal domain, F3 (amino acids 198-314) in the pET28b expression system. We vaccinated mice with the NH36, or each one of the peptides and saponin and challenged them with *L. (L.) infantum chagasi or L. (L.) amazonensis* (9).

Thus, we described for the first time the role of the nucleoside hydrolases in the induction of immunoprotective CD4^+^ T cell driven or CD8^+^ T cell-mediated cytotoxic immune response, in the mouse model of visceral and cutaneous leishmaniasis (9). We identified that, in the mice challenged with *L. (L.) infantum chagasi*, the adaptive immune response of mice is directed against the F3 domain and is mainly mediated by CD4^+^ T cells with a minor contribution of CD8^+^ T cells (9). Actually, immunization with the F3 peptide exceeded the protective efficacy promoted by the cognate NH36 protein by 36% (9, 103). The increases in DTH and TNF-α/IL-10 ratios were the strong correlates of protection in the F3-vaccinated mice, and were negatively associated to the 88–90% of reduction of the parasite load. Additionally, increases in IgG1 and IgG2 anti-NH36 antibodies, CD4^+^ T cell proportions, secretion of IFN-γ, ratios of IFN-γ/IL-10 producing CD4^+^ and CD8^+^ T cells and percentages of inhibition of binding of anti-NH36 antibodies to predicted epitopes were also detected, in mice vaccinated with F3. Vaccination with the F1 peptide, on the other hand, although induced an immune response, did not show any impact on the *L. (L.) infantum chagasi* parasite load (9).

In contrast, both the F1 and F3 domains reduced the size of footpad lesions of vaccinated mice infected with *L. (L.) amazonensis* (9). Our results therefore suggested that the domains of NH36 could be the basis for the development of a bivalent vaccine against visceral and cutaneous leishmaniasis (9). Both F1 and F3 vaccines enhanced the IgG and IgG2a antibodies and the F3 promoted the strongest DTH and the highest proportions of CD4^+^ and CD8^+^ NH36-specific T cells and secretion of IFN-γ and TNF-α. According to experiments of depletion with monoclonal antibodies, the cross-efficacy against the infection by *L. (L.) amazonensis* is based on a CD4^+^ T cell-driven response against the C-terminal domain that determines a 75% reduction of the lesion sizes, and on a CD8^+^ T cell response against the N-terminal domain of NH36, that reduced the lesions by 57% (10). The F3 alone (9) or together with the F1 vaccine (9, 10) induced durable protection against visceral and cutaneous leishmaniasis, respectively, in the mice model. This cross-protection was explained by the 93% of homology between the amino acid sequence of *L. (L.) donovani* NH36 and the NH A34480 of *L. (L.) amazonensis* (10). Both shared conserved predicted epitopes for CD4^+^ and CD8^+^ T cells in the F1 domain, and of CD4^+^ epitopes diverging in only one amino acid, in the F1 and F3 domains (10).

The F3 and F1 vaccines were also effective in the immunotherapy of mice infected with *L. (L.) amazonensis* and reduced the parasite load and the sizes of lesions (11). Interestingly, the strongest antibody and DTH response against *L. (L.) amazonensis* was promoted by the F3 peptide, which also induced a Th1 cytokine response, with increased levels of IFN-γ and TNF-α but reduced levels of IL-10. In contrast, the F1 vaccine, induced no DTH and promoted similar IFN-γ, TNF-α and IL-10 secretion, suggesting the presence of regulatory T cell epitopes in its sequence (10). In the case of the F3 vaccine, the efficacy advanced as the frequencies of the CD4^+^ T cells changed from single (IL-2^+^, IFN-γ^+^ or TNF-α^+^) to double-cytokine secretors (IL-2^+^TNF-α^+^, TNF-α^+^IFN-γ^+^, and IL-2^+^IFN-γ^+^). In contrast, the F1 vaccine induced, as described before (10), a main advanced CD8^+^ T cell response that changed qualitatively from single to triple-cytokine secretors (IL-2^+^TNF-α^+^IFN-γ^+^), the cell phenotype involved in memory immune responses (104). Therefore, the cure of cutaneous leishmaniasis depends on the generation of a Th1 response by the F3 vaccine and a CD8^+^ T cell response, by the F1 vaccine (11).

## Optimization of the Prophylactic Efficacy Presenting the Immunogenic Domains as a Recombinant Chimera

The theory that defends the development of T-cell epitope vaccines considers that the whole protein antigen is less potent for vaccination than its fractions that are composed of the relevant immunogenic epitopes (93, 103). We have confirmed that for the NH36 vaccine (9–11). These relevant domains would also be more potent than the isolated epitopes. For the optimization of the vaccine efficacy, we should therefore use the domains, either admixed or expressed in tandem as one recombinant chimera. In the case of the chimera, the domains epitopes could be simultaneously presented by the MHC Class II and Class I receptors of a single APC to CD4^+^ and CD8^+^ lymphocytes, respectively, by cross-presentation. This possibility could also potentiate the vaccine efficacy. Recent investigations pointed out the importance of the promotion of both CD4^+^ and CD8^+^ multifunctional T cells for protection against leishmaniasis (89, 90, 104).

Moreover, from the vaccine manufacturer's point of view, the expression and purification of a licensed single recombinant chimera vaccine including the two domains, would be easier to achieve and more economic, than the expression and isolation of each one of the domains individually (12).

In order to check these possibilities we compared the prophylactic efficacy of the NH36, F1, F3, admixed F1+F3 domains and the F1F3 recombinant chimera in mice, against the infection *L. (L.) amazonensis* (12).

While the chimera promoted the highest anti-NH36 antibody levels, and the strongest DTH and the highest secretions of IFN-γ and IL-10, the F3 vaccine induced the highest secretion of TNF-α (12). Furthermore, the chimera and the F1 vaccine also stimulated the increase of the frequencies of CD4+ and CD8+ T cells secreting IL-2, TNF-α or IFN-γ alone, or any combination of two cytokines. In addition, the F1 and chimera vaccines enhanced the frequencies of the CD4+ multifunctional cells secreting IL-2, TNF-α and IFN-γ simultaneously (12), which are the type of cell correlated with the desired Th1 memory response (89, 104). The admixed domains and the chimera reduced the skin lesions sizes by 80 and 84% respectively. In addition, the chimera generated the most pronounced and significant reduction of the parasite load (99.8%). We concluded that the epitope presentation by the recombinant chimera F1F3 optimizes the immunogenicity and efficacy of the vaccine above the levels induced by the independent or admixed F1 and F3 domains (12). The chimera vaccine also induced a Th1-CD4^+^ T cell response that targeted the FRYPRPKHCHTQVA epitope of F3 and the YPPEFKTKL epitope of F1, which together with DVAGIVGVPVAAGCT, FMLQILDFYTKVYE and ELLAITTVVGNQ, also induced the strongest CD8^+^ T cell response and secretion of IL-10. Therefore, the YPPEFKTKL sequence might promote the CD4^+^, CD8^+^ T cell, and a potential T regulatory response. These results represented an important step toward the design of a nucleoside hydrolase NH36 based polytope vaccine for the generation of cross-protection against cutaneous leishmaniasis (12).

## Toward the Design of a Multi-Epitope Human Vaccine Against Leishmaniasis

The results disclosed by our investigations about the potential use of the NH36 antigen in diagnosis and vaccination against leishmaniasis (5, 9–12, 53, 56, 77, 98–101) has inspired the work of other groups, which have used the NH36 recombinant protein for the development of a formulation against human visceral leishmaniasis (15, 94, 95). The whole NH36 of *L. (L.) donovani* and the sterol 24-c-methyltransferase (SMT) from *L. (L.) infantum* were expressed as a fusion protein called LeishF3 that is being tested in clinical trials (15, 105). When formulated with glucopyranosyl lipid A-stable oil-in-water nanoemulsion (GLA-SE), a Toll-like receptor 4 TH1 (T helper 1), the Leish+F3 protein protected mice against *L. (L.) donovani* and *L. (L.) infantum* infections, showing multifunctional CD4 T cells secreting IFN-γ, TNF-α and IL-2 with low levels of IL-5 and IL-10. This protection was mediated by CD4^+^ but not by CD8^+^ T cells (15). These results confirmed our previous data obtained after vaccination with the NH36 antigen in mice (9, 24) and dogs (101). In fact, the NH36 vaccine induced protection against *L. (L.) infantum chagasi*, which was depleted with the treatment with anti-CD4^+^ antibody (9).

Furthermore, a phase 1 clinical study with LeishF3 +GLA-SE was performed in healthy, uninfected adults in the United States. LeishF3, with or without the GLA-SE adjuvant, was tested in 36 healthy adults volunteers (15). The vaccine was immunogenic and well-tolerated. Only tenderness and/or pain at the injection site, fatigue and decreased hemoglobin were reported as adverse effects. As expected for the use of an adjuvant that contains QS21 saponin, an increased TH1 and TH2 cytokine response was observed in individuals that received LeishF3+GLA-SE. The vaccine was immunogenic when administered with adjuvant and increased mainly the IgG1 and IgG3 antigen-specific subtypes of antibodies in serum. In addition, the secretion of IFN-γ, TNF-α, IL-2, IL-5, and IL-10 in response to Leish+F3 was enhanced in vaccinated subjects. However, this was followed by a decrease in IL-10 and IL-5 levels and sustained levels of IL-2 (against NH36 and SMT) and TNF-α (against NH36) (15, 104). These results confirm our previous descriptions of the induction of strong IL-2 and TNF-α antigen specific responses in mice vaccinated with NH36 and its domains (9–12, 106) and in human asymptomatic DTH+ and cured individuals infected with *L. (L.) infantum chagasi* of Brazil (13) and *L. (L.) infantum* of Spain (14).

We demonstrated, in the mice model, that the efficacy of the NH36 vaccine can be optimized by using the correct mixture of domains and epitopes (9–12). In the development of a multi-epitope vaccine destined to protect against cutaneous and visceral human leishmaniasis, two priorities should be taken into consideration. The selected epitopes and domains should be conserved among the different *Leishmania* species (13), and the epitopes should be highly promiscuous and bind to as many allotypes of HLA DR, DQ and DP MHC Class II, and of HLA A and B MHC Class I histocompatibility genes and T cell clones as possible (97).

Aiming to identify the main domains and epitopes of NH36 that deserve to be included in an optimized vaccine, we recently assessed in cured and active patients of visceral leishmaniasis and in asymptomatic infected subjects from Brazil their cytokine secretion, in response to NH36 and its domains, and their clinical symptoms (13). The F2 and F1 domains constituted markers of resistance to VL, since together they promoted the highest secretion of IL-17, IL-6 and IL-10 in asymptomatic DTH^+^ individuals. In addition, F2 also provoked the strongest secretion of IFN-γ, TNF-α and IL-1β and the highest proportions of CD4^+^ T cells secreting IL-2^+^, IL-2^+^TNF-α^+^, IL-2^+^IFN-γ^+^ and IL-2^+^TNF-α^+^IFN-α^+^, both in cured and asymptomatic subjects. In agreement, in response to F1, the IFN-γ increase was correlated with decreased sizes of liver and spleen and with increased hematocrit counts. Moreover, in response to the F2 domain, the IFN-γ increase was associated to an increased hematocrit and hemoglobin counts (13). Furthermore, the enhancements in IL-17 secretion correlated also with decreased spleen and liver sizes in response to F1 and F2.

In contrast, the F1 and F3 domains amplified the frequencies of CD8^+^ T cells secreting IL-2^+^, IL-2^+^TNF-α^+^, and IL-2^+^TNF-α^+^IFN-γ^+^ of patients with active VL, in association with the enlargement of spleen and liver sizes, and decreased hematocrit and hemoglobin counts. We concluded that the cure and acquired resistance to VL is associated to the CD4^+^Th1 and Th17 T-cell responses to the F2 and F1 domains. In contrast, clinical signs of VL correlate to CD8^+^ T-cell responses against F3 and F1. In agreement with our experimental results, the initial *in silico* prediction with the TEPITOPE software disclosed one epitope in F1 and two epitopes in F2 that bind to at least 19 of 25 HLA-DR allotypes of MHC Class II molecules. In addition, five epitopes for CD8^+^ T cells were disclosed in F1 and F3 by the SYFPEITHI software. These epitopes bind to at least 17 HLA-A and B allotypes (13). All these epitopes were highly conserved in species of the *Leishmania* subgenus, and showed minor variations in the *Viannae* subgenus. The *in silico* predictions were confirmed since the three synthetic epitopes predicted for the CD4^+^ T cells in F1 and F2 as well as two epitopes of F1, predicted for CD8^+^ T cells stimulated a significant secretion of IFN-γ by PBMC in asymptomatic DTH^+^ *L. (L.) infantum chagasi* infected subjects. Since no human vaccine against *Leishmania* is available so far, our results indicate that the identified domains and epitopes of NH36 could be used in combination to optimize a universal multi-epitope vaccine against leishmaniasis (13).

In addition, the antigenicity of the NH36 domains was tested on *L. (L.) infantum* infected subjects from Spain (14). The F1 induced a 19 % stronger PBMC proliferative response than NH36, in asymptomatic infected subjects. Moreover, F1 promoted a 42–43% higher IFN-γ and TNF-α secretion in cured subjects. In addition, higher IL-17 secretion was promoted by the F1 in asymptomatic subjects, and by NH36 in cured cutaneous leishmaniasis patients. Furthermore, as predicted *in silico*, a Granzyme B increase was detected in supernatants from cured patients after stimulation with either NH36 or F1 [13, 14].

Regarding the antibody response, F1 and NH36 differentiated the IgG3 response in *L. (L.) donovani* patients with VL from Ethiopia and *L. (L.) infantum* patients from Spain. In contrast, NH36 recognized with higher titers the sera from *L. (L.) donovani*-infected individuals from Ethiopia, suggesting species-specificity. Therefore, the F1 domain, which induced a Th1 and Th17 response in cured and DTH^+^ *L. (L.) infantum chagasi* infected subjects, may also promote a protective immune response against *Leishmania (L.) infantum* (14).

## Conclusions

Nucleoside hydrolases are vital enzymes for the replication of *Leishmania*, conserved phylogenetic marker of the genus and strong-specific immunogens. We demonstrated that NH36 is an excellent target for chemotherapy of visceral leishmaniasis. Searching for the most immunogenic fraction of promastigotes of *Leishmania* we described the FML antigen of *L. (L.) donovani*, that has as its main component, the NH36 Nucleoside hydrolase. We developed second–generation vaccines with the FML and the NH36 native antigens, and with the NH36 recombinant protein. In addition, we obtained a third generation vaccine based upon the NH36 gene. All these vaccines were protective and curative when assayed in the murine and canine models of leishmaniasis, and the FML-vaccine called Leishmune® reduced the incidence of human and canine disease in the endemic areas. Recently we initiate the search for the most immunogenic part of the NH36 sequence aiming to optimize the vaccine efficacy. We observed that the F3 vaccine has an important role in signaling the optimal control of visceral leishmaniasis in mice and that the F1 domain is also needed for protection against tegumentary leishmaniasis. This knowledge allowed us to develop a recombinant chimera vaccine, which optimized the vaccine efficacy. Furthermore, aiming to develop a potential T-cell epitope synthetic vaccine against both human visceral and cutaneous leishmaniasis we identified the major immunogenic regions of NH36 recognized by PBMCs of cured and asymptomatic patients infected with *L. (L.) infantum chagasi* in Brazil and *L. (L.) infantum* in Spain and *L. (L.) donovani* in Ethiopia. In each step of our investigation the vaccine formulation including NH36 and its domains gained in efficacy and safety. Once all the major HLA DR, DQ and DP and HLA-A and B promiscuous epitopes of NH36 have been identified, the design of a multi-epitope vaccine for prevention of human visceral and cutaneous leishmaniasis will be completed.

## Author Contributions

The author confirms being the sole contributor of this work and has approved it for publication.

## Conflict of Interest Statement

CP-d-S is one of the inventors of the patent file PI1015788-3 (INPI Brazil) owned by the Federal University of Rio de Janeiro.
